# The modern gut-hammer: Understanding the eating habits of loggers through Photovoice

**DOI:** 10.1016/j.appet.2021.105882

**Published:** 2021-12-24

**Authors:** Judy Graham, Erika Scott, Pam Tinc, Liane Hirabayashi

**Affiliations:** Northeast Center for Occupational Health and Safety in Agriculture, Forestry, and Fishing, Bassett Medical Center, One Atwell Road, Cooperstown, NY, USA

**Keywords:** Logger, Diet, Photovoice, Occupational Health

## Abstract

“Give Us Your Best Shot” is a Photovoice project designed to shed a light on loggers’ food choices, attitudes toward and challenges around eating. This research focused on answering the question: What do you typically eat on a workday and where do you get it (breakfast, lunch, dinner, snacks, and drinks)? Consider: What makes it hard to eat healthier? If you feel you eat healthy, how do you do it? Participants were asked to take a photo to answer our research question at least once a week for a six-week period in autumn 2019. Photos and comments were exported from REDCap and imported into NVivo 12, a qualitative analysis software. Two members of the research team analyzed these data. In total, six male Maine loggers, ages 33 to 64, took part in the Photovoice project. Several themes emerged from these data including, but not limited to, the conflict between stated feelings about diet and health and actual consumption habits, the priority of health among many demands, and perceived healthfulness. Data analysis revealed time and family to be significant influential factors affecting loggers’ attitudes and ability to eat healthfully.

Modern trends toward processed and pre-made food resulting in less home cooking, impacts how and what loggers eat. This project served to show that food choice and diet are modulated by complex outside forces and that improving diet is not a straightforward task. Maine loggers are coping with the same struggles that many workers face, with the added hardship of dealing with extremely long work hours and commutes, leaving little time for anything else. These factors should be taken into consideration when planning any nutrition related interventions with logging workers.

## 1. Introduction

For generations the ‘gut-hammer’ called lumberjacks to gather for meal time deep within the woods (Davis, 1942). Back then, the work demanded that logging crews spend long stretches working a plot of land, and sleeping quarters and cookhouses were commonplace in logging camps. As land-use and socioeconomic factors have shifted in the northeast over the last 140 years from homestead agriculture to manufacturing focused communities, so have trends in what and how people eat (Lindsey et al., 2013). Food traditions are based on a variety of factors surrounding how people eat (timing, environment, social interaction, food appreciation versus wasting and concerns of quality and quantity). They are also based on what people eat including available ingredients, processing preparation, timing and variety (Sproesser et al., 2019). Historically, into the mid-20th century, a variety of grains, dairy, livestock, potatoes, vegetables and fruit were produced on small family farms in Maine (Robin Beck NC et al., 2011). Early Mainers ate foods available year round including meat, eggs, dairy, and foods that could be preserved through drying, smoking or fermenting process. These included beans, beets, squash, apples and blueberries. In the summer months, a variety of leafy green vegetables, squash and snap peas were grown in gardens. In colder seasons, Mainers ate the foods that could be cold stored or preserved. Prior to home refrigeration, access to leafy greens and softer fruits and vegetables not amenable to preservation were mostly limited to summer months. Mutton was a main staple, but diets also included pork, beef, fish and chicken (Robin Beck NC et al., 2011). Logging outfits and their company cook houses of 1910 and on in Maine had access to these ingredients and more; they supplied loggers not only with needed calories but with a plethora of available whole foods and baked goods as a type of enticement for company loyalty (Conlin, 1979). Company cookhouses were the ultimate in benefits packages for early loggers. Sample menus included a staggering quantity and variety of food, but also a quality of food which at the time was remarkable. In Maine, the shift away from an agrarian society to a manufacturing based economy in the 1900s, where most food was produced and eaten at home, changed the way people ate. Overall research has shown trends in US home food preparation have been dropping significantly since 1965 and that only about half of US adults spend any time partaking in daily cooking activities (Lindsey et al., 2013). Compounding this change was the eventual shift away from historical logging traditions of cook camp menus to shift work and mechanized logging of the later 20th Century. Today, loggers travel long distances to the wood lot, but most return home to sleep (Scott et al., 2020). In some ways, the typical loggers’ diet and lifestyle may look similar to that of their grandparents or great-grandparents, but in others, it is dramatically different.

Despite many safety improvements made in the last decades, logging is one of the most hazardous industries in the United States (US), On a typical day in the life of a logger, workers operate complex machinery, experience unpredictable and extreme physical forces and weather conditions, and navigate rugged terrain and often high working platforms. Workdays are very long and often in remote locations. Loggers are injured or killed every year by falling objects such as trees and limbs, falling from high surfaces, or being struck by hand-held equipment or vehicles. In 2019, the fatal injury rate for logging workers was 68.9 per 100,000 full-time equivalent (FTE) workers, which is twenty times higher than the all-worker fatality rate of 3.5 per 100,000 FTE (Bureau of Labor StatisticsInjuries, 2019). The rate of non-fatal work injury and illness in 2019 was 208.7/100,000 FTE compared to 86.9 for all private industries (Janocha & Hopler, 2018). While significant, injury and death are not the only risks loggers face on a daily basis.

In an extensive health study conducted by the Northeast Center for Occupational Health and Safety in Agriculture, Forestry, and Fishing (Northeast Center) from late 2018 to mid-2020, a cohort of over nearly 400 Maine loggers shared details about their lives ranging from work conditions to personal health information to the impact of COVID-19 on their operations. The study entailed seven surveys spaced at threemonth intervals and a health questionnaire. In addition, over 80 loggers participated in an extensive physical health assessment, which included weight, height, waist-hip measurements, blood pressure, respiratory peak flow, carbon monoxide levels, blood glucose and cholesterol, Framingham Risk scores, audiometry testing and vision screening, as well as an extensive physical exam. Loggers reported working close to 12 h a day, with the majority starting their workday well before 6:00. In addition to their long workdays, conventional loggers had an average one-way commute of 41 min with mechanized loggers traveling even longer, on average 70 min, to get to work (Scott et al., 2020). Based on the health assessment, we found that 45% of loggers had blood pressure consistent with stage II hypertension, with another 30% in stage I hypertension. The median body mass index (BMI) score was over 30, in the obese category.

### 1.1. Food is an important component of health

Like all workers, loggers need proper nourishment to maintain health and energy for demanding daily activities. For loggers in particular, it is extremely important to maintain a sharp focus while operating logging equipment and tools in inherently dangerous environments. There is a strong connection between mental health, wellbeing and dietary patterns. Research has shown that proper nutrition and physical interventions in workplace settings improve health factors and decrease the rate of absenteeism (Grimani et al., 2019; Hong & Peltzer, 2017). Diet patterns imbue long-term health consequences, as research has shown that consistent diet patterns as opposed to nutritional intake are most important to long-term health outcomes. Some diet patterns, such as the Mediterranean diet—also known as the “prudent diet”—are shown to be highly reliable for controlling risk associated with cardiovascular disease (CVD) and non-cancer, non-cardiac inflammatory diseases (Jacobs & Tapsell, 2015).

Consistent, adequate nutrition and caloric intake is important not only for normal metabolic function but also for immune system function. Diets rich in processed food—high in carbohydrates, fats, and salt—have been linked to cytokine expression and inflammatory response, as well as immune system dysfunction (Myles, 2014). Data also show that diets high in fat, sodium and calories greatly contribute to risk factors that affect health, specifically cardiac health (Ferdinand & Mahata, 2017). High low-density lipoprotein (LDL) levels are also known to have inflammatory effects on arterial walls and may lead to atherosclerosis (hardening of arteries) and coronary artery disease (CAD) (Hansson, 2005). As a result of consistent, low-quality diets, other biomarkers for stress, including high sensitivity C-reactive proteins, are also known to be elevated, which has been shown to lead to arterial stiffness and CVD (Ferdinand & Mahata, 2017). Eating fast food two or more times per week is associated with over a 50% increase risk of coronary heart disease death over no fast-food intake; and this risk is linear based on frequency of consumption (Saluja et al., 2021).

Loggers need to get through the workday but also live long, healthy productive lives, and diet patterns are integral in achieving this. The presence of increased cardiac risk factors among this cohort was the impetus for “Give Us Your Best Shot,” a Photovoice project designed to shed light on loggers’ food choices, barriers, and attitudes toward eating.

### 1.2. What is Photovoice

The purpose of this research was to explore the diet and attitudes towards food among Maine loggers using a qualitative approach called Photovoice. Used to understand many public health phenomena, including dietary habits (Neill et al., 2011; Yankeelov et al., 2015), Photovoice captures the participant’s voice and vision directly through words and pictures. Social scientists have been using Photovoice for decades as a qualitative analysis tool to help empower communities for change (Wang, 1999). The intention of this exercise was to give voice to a relatively small and hard-to-reach group: to hear their thoughts about their health and eating habits and compare that with what we know from the quantitative health study as well as to evaluate the feasibility of the Photovoice method with this cohort. Research has shown that Photovoice projects with relatively few participants still provide a unique ability to gather descriptive and visual data that is relevant and important for change (Catalani & Minkler, 2010). Prompted by the research completed with Maine loggers in 2018, “Give Us Your Best Shot” provided unique insight into a highly specialized workforce and has added to overall understanding of important health risks outside of the physical workspace.

## 2. Methods

### 2.1. Questions asked

This Photovoice project focused on answering the questions: What do you typically eat on a workday and where do you get it (breakfast, lunch, dinner, snacks, and drinks)? Consider: What makes it hard to eat healthier? If you feel you eat healthfully, how do you do it?

### 2.2. Recruitment

The study team developed a recruitment flyer and brochure explaining the purpose of the study, what was involved, and how to signup. These materials were distributed using the project’s Facebook page for the existing occupational cohort study of Maine loggers (@Main-eLoggerHealthSafetyStudy), and in person at the Woodmen’s Field Day in Fryeburg, Maine, in September 2019. Participants had to be 18 or older, plan to earn at least half of their income from logging in the year ahead, have a smartphone or camera, and be willing to share their photos and comments with the research staff. We aimed to enroll between five and ten participants. Informed consent was obtained from each participant and project instructions were distributed. These instructions outlined the topic for photos, due dates for photos/comments, and guidelines for photo submission. These guidelines included taking photos at a variety of locations like the job site, commute, logging company/garage, home, places of recreation, or eateries. Participants were asked to maintain the privacy of others and not include other people’s faces or recognizable features in photos. Participants were urged to prioritize safety and not take photos when it could put themselves or others in harm’s way (e.g., driving, operating equipment). Participants received an incentive of $50 L.L.Bean Gift cards at the beginning of the study, and an additional $50 L.L.Bean gift card at the conclusion of their evaluation.

### 2.3. Data collection

Participants were asked to take a photo to satisfy our research question at least once a week for a six-week period in autumn 2019. These photos were transmitted to the research team via text message to a study cell phone or email. Comments were included with these photos either by text or voice memo. Once received, these data were entered into a REDCap database by study staff. After the six-week period, participants had a debrief/interview phone call with a study staff member. Participants were asked ten questions relating to their experience with the Photovoice project. Two questions related to how much they liked participating in the project. Two questions related to their attitudes around eating, including how they prioritize their own diets and nutrition and if they noticed any patterns in their own photos. Loggers were asked to look at their favorite photo and answer four questions about what they saw, what was happening in the photo, how it related to their lives, and what they might do differently. Participants were also asked what they considered the important issues loggers are facing that researchers should know about. Responses were transcribed, imported into NVivo and coded for analysis as described in section 2.4. Analysis of quality and consistency of data entry into REDCap was performed by the research coordinator and/or principal investigator, and data entry protocols were revised where necessary to ensure the fidelity of these data.

### 2.4. Data analysis

Photos and comments were exported from REDCap and imported into NVivo 12 (QSR, Queensland, AUS), a qualitative analysis software. In addition, staff described the contents of each of the photos and imported the document into NVivo. These data were analyzed by two members of the research team using a content analysis approach. Photos, participant comments, content descriptions and evaluation data were included as files for analysis. Figure titles in subsequent sections include direct quotes from the loggers. Nodes were created both inductively and deductively, and two coders reviewed these data as a team to evaluate and discuss themes. A third qualitative researcher reviewed and added to the NVivo analysis, as well. Nodes (meat, vegetables, junk food, homemade lunches, store bought lunches, time, work, etc.) were created based on text queries and word frequencies and other NVivo tools including word clouds and hierarchy charts to help visualize patterns and prioritize responses. Relationships between emerging ideas were further connected using NVivo code analysis. Themes were later developed from ideas and attitudes that emerged from the data. Themes relating to time, health, family, and stress were further developed when comparing similarities and differences among participants.

The Institutional Review Board (IRB) of the Mary Imogene Bassett Hospital reviewed and approved this research protocol.

## 3. Results

In total, six male Maine loggers, ages 33 to 64, took part in the Photovoice project. Several themes emerged from these data including, but not limited to, the conflict between stated feelings about diet and health and actual consumption habits, the priority of health among many demands, and perceived healthfulness. Data analysis revealed time constraints imposed by work conditions and cultural and traditional factors as well as family interactions to be significant influential factors affecting loggers’ attitudes and ability to eat healthfully.

### 3.1. Summary of eating habits

**Fig. 1.**
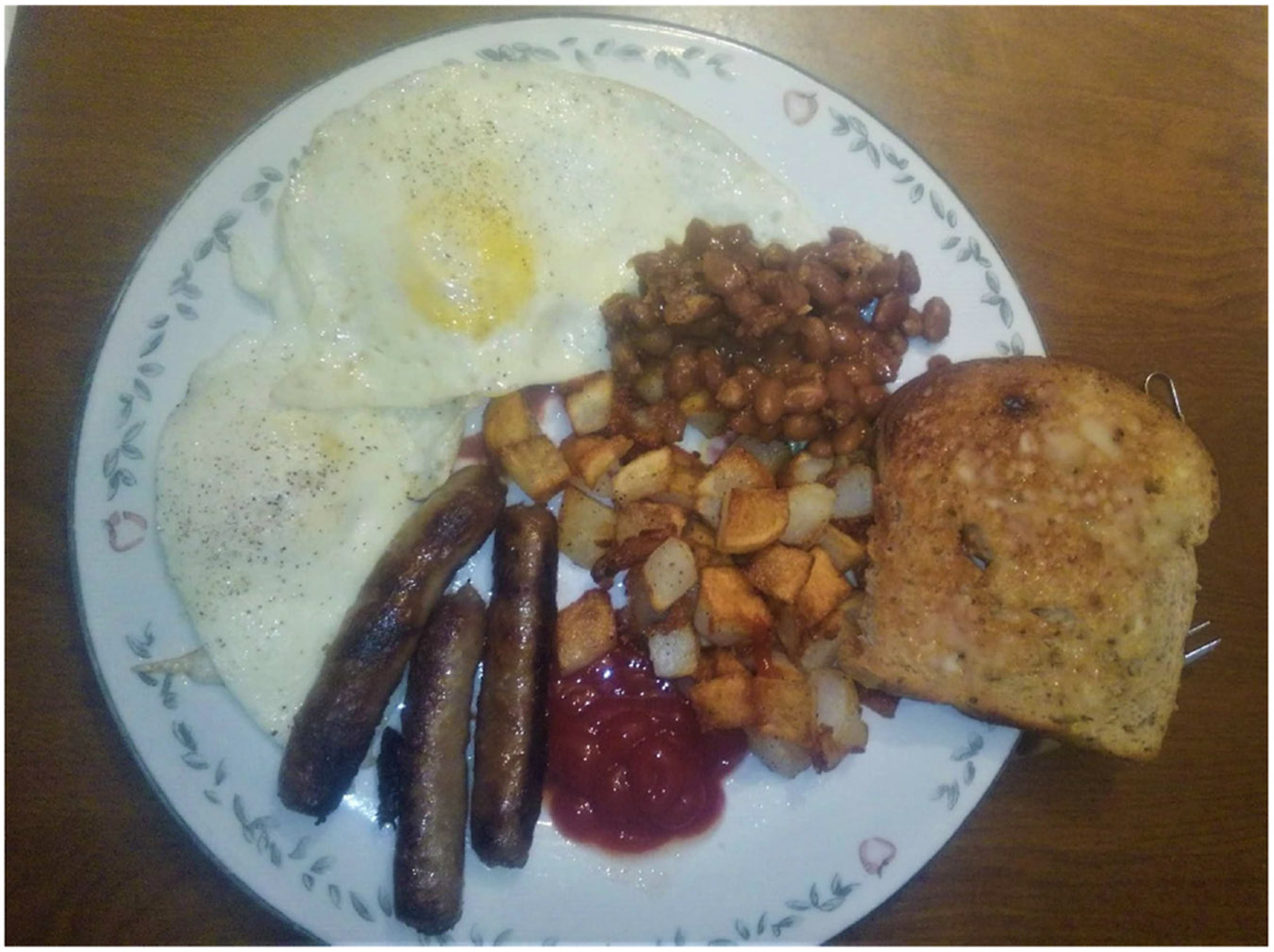
“How’s that for a nice breakfast! My wife’s good to me as you can see I have beans just like the loggers from years ago. You got to start the day out right!” (PID 303).

The meals documented through Photovoice varied widely: They ranged from homemade meals, to convenience store pizza, to commercial diet shakes and highly caffeinated energy drinks. Overall, the diet patterns and descriptions fell into two general categories: (1) a diverse representation of relatively healthy food that sparked feelings of pride, and (2) fast food and low calories (diet) with feelings of regret. The loggers who prepared meals at home seemed to indicate a belief that the meals were of high quality and provided needed energy for the day’s work, while those who purchased meals commented on poor quality food and bad choices. Other negative feelings related to the need to adhere to a calorie-restricted diet because of health concerns. This may be reflective of several themes: differences in the physical demands of modern logging, health concerns of participants, and/or socioeconomic, traditional or cultural factors. Homemade breakfasts were almost all prepared with eggs, sausage, beans, toast, coffee and juice with the exception of the calorie restrictor who consumed packets of flavored pre-packaged oatmeal. Participants harkened back to traditional logging meals referring to *“the days of old”* (PID 303) (Fig. 1). The meal shown in this photo consists of readily available processed foods (sausage, beans, bread) and two whole foods (eggs and potatoes). This meal is reminiscent of the traditional logging camp staples of beans, bread and sausages and consists mostly of protein and carbohydrates, has little soluble fiber content and is presumably high in salt and fat content.

**Fig. 2.**
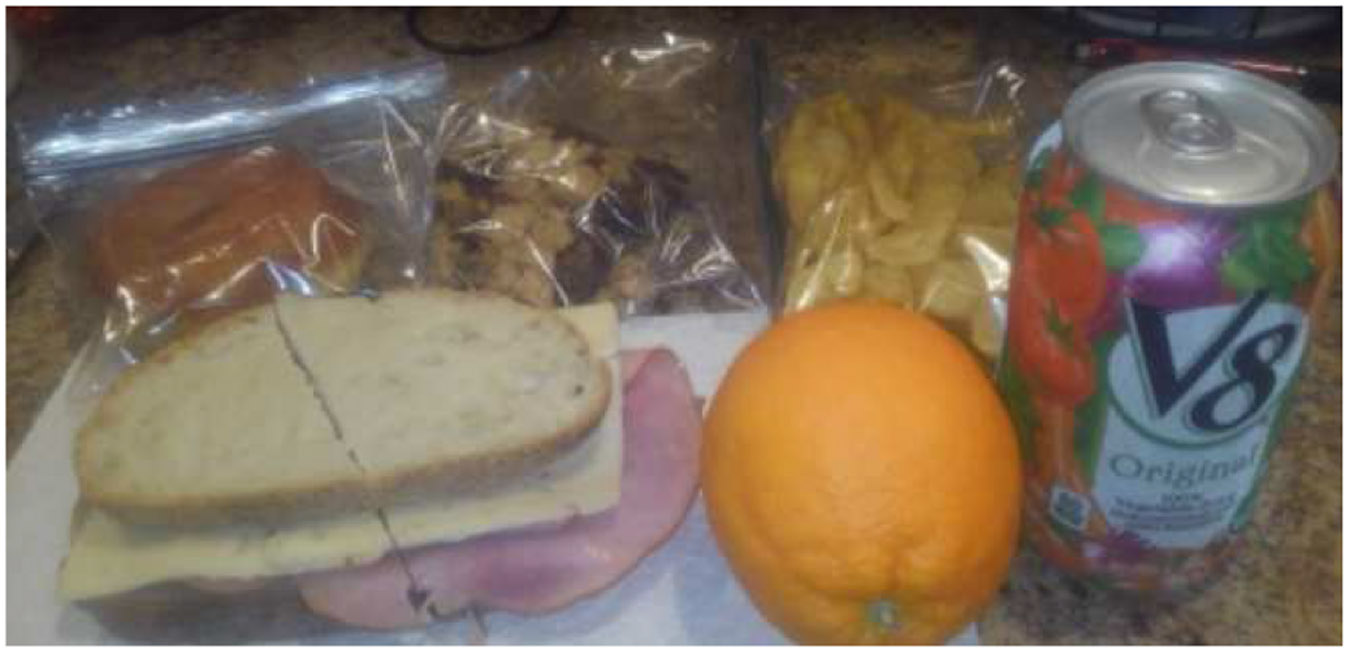
“Healthy lunch with carbs fruit and V8 instead of soda” (PID 303).

Two participants indicated that they consistently prepared their own lunches, which had a great variety in form and generally included sandwiches made with deli meats, fruits, yogurt cups, cookies, potato chips and non-carbonated drinks (Fig. 2). Although in a variety of forms, the foods in this photo consist largely of carbohydrate sources including the manufactured donut, chips, granola, and bread. Protein sources are provided through processed meat and cheese slices while some other nutrients as well as carbohydrates are provided in the bread, V8 beverage and orange. The participant notes the carbohydrate sources in the food and points out the substitution of V8 for soda.

Participants who prepared lunches at home were the same participants who submitted elaborate home-cooked breakfast photographs, and who appear to have a more varied diet pattern and better quality nutrition than the other Photovoice participants.

**Fig. 3.**
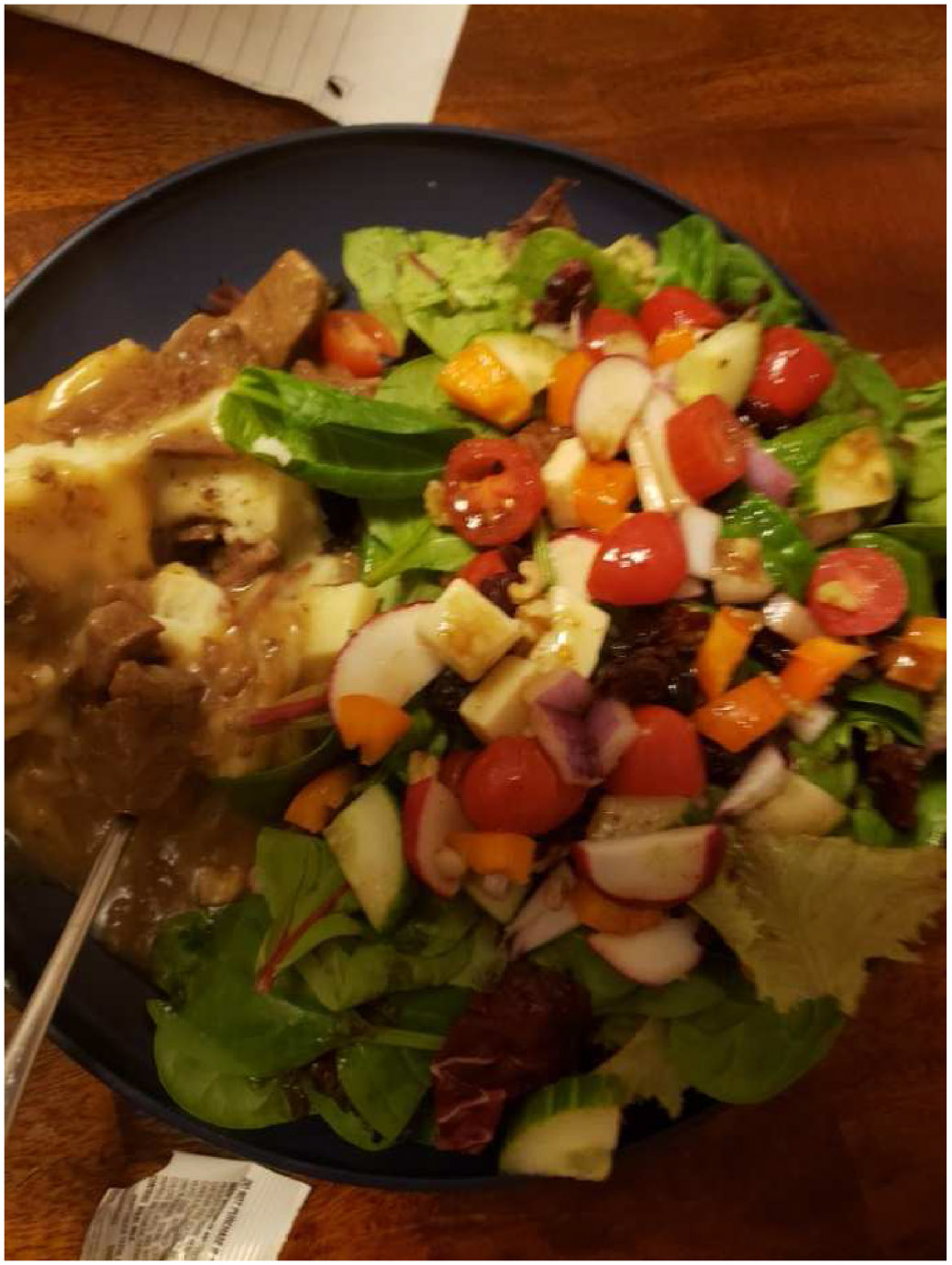
“Meal consists of over half plant based diet!” (PID 1827).

One participant occasionally brought leftovers from home and these meals were made with ground venison with tomatoes and rice. Dinner photographs all highlighted homemade meals and tended to consist of meat (often venison or pork), a starch (rice or potatoes) with a green vegetable. Only one meal included salad, which was consumed as part of calorie restriction plan (Fig. 3). The photo is one of the few that had any leafy greens and fresh vegetables included that would provide variety of nutrients and soluble fiber. Although hidden under the salad, there is a portion of roasted meat and possibly gravy that provides protein and fats. This participant indicated that he is trying to eat healthier because of health issues and quality of life wishes, but one gets the sense that he is struggling with his diet overall in terms of calories and satiety.

**Fig. 4.**
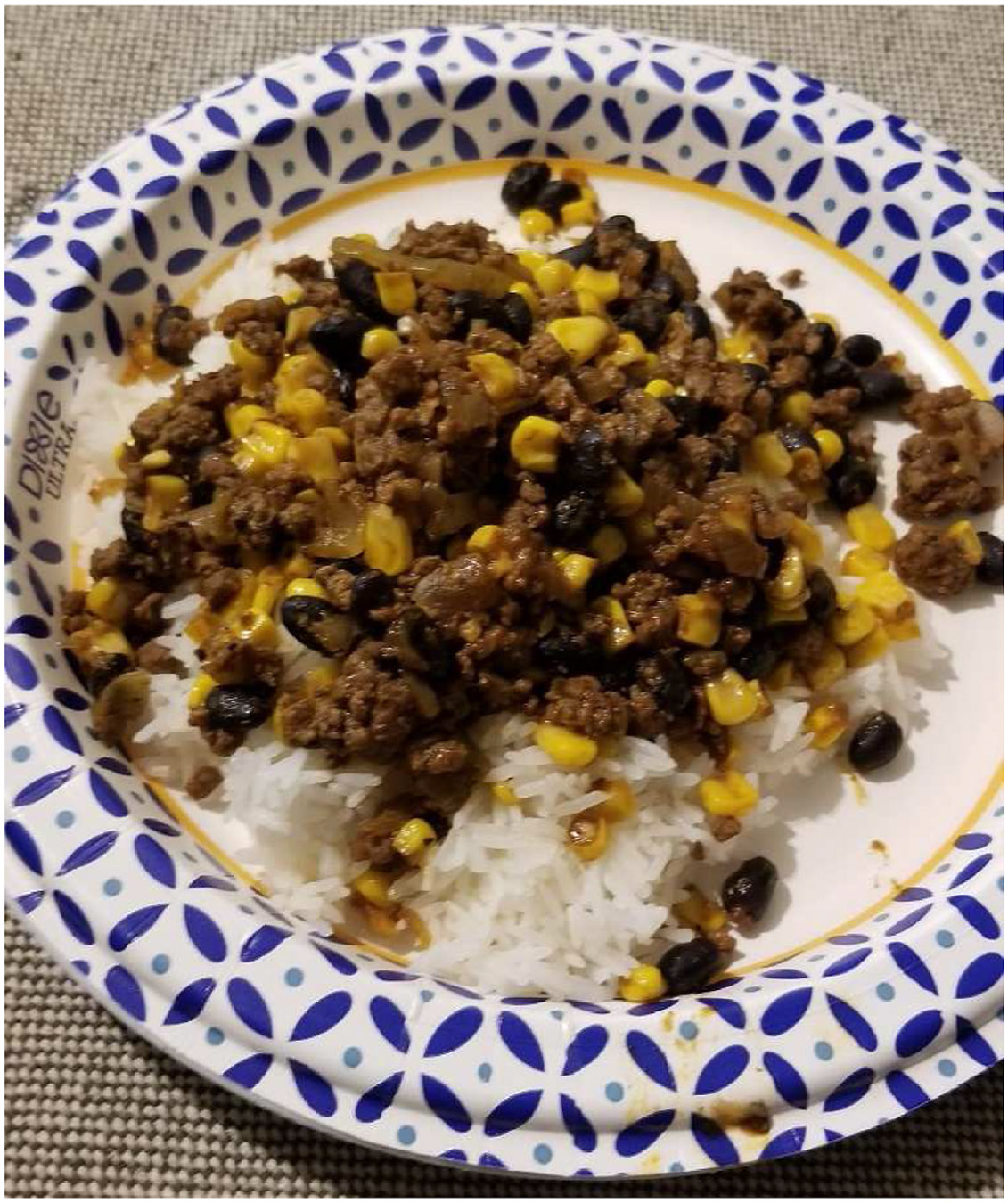
Quote “This is a meal I cook for a few days and do a lot with the leftovers. It’s venison hamburger with black beans and corn with some Mexican style spices. You can put it over rice, hash browns, or anything else you want to go with it” (PID 16).

Some loggers expressed that they focus on meal preparation. One participant described snack preparation that was a hybrid of store-bought and homemade, adding healthy fruit to yogurt in single serve containers, saying, *“Snacks like these make it easy to have a healthy snack at work. We buy it in a large container and put it in Tupperware with fruit mixed in. Easy to put together and bring [to] your work” (PID 1828).* Another participant highlighted the importance of natural meat sources (venison) and batch cooking to make multiple meals at once (Fig. 4). Food depicted shows a homemade dish with venison harvested by the participant. It includes white rice, black beans and corn providing source of carbohydrate and some fiber. Although lacking in nutrients that might be provided through dairy, leafy green vegetables and fruit, the ingredients are of generally higher quality and less processed than store bought meals.

Photographs depicting convenience store foods tended to be relatively high in protein, carbohydrates, fats, and salt. They appeared extremely low in fiber, with very few fresh vegetables and fruit. Store-bought lunches included pizza and cheeseburgers, energy drinks, corn and potato chips, pre-packaged muffins, or submarine deli type sandwiches).

**Fig. 5.**
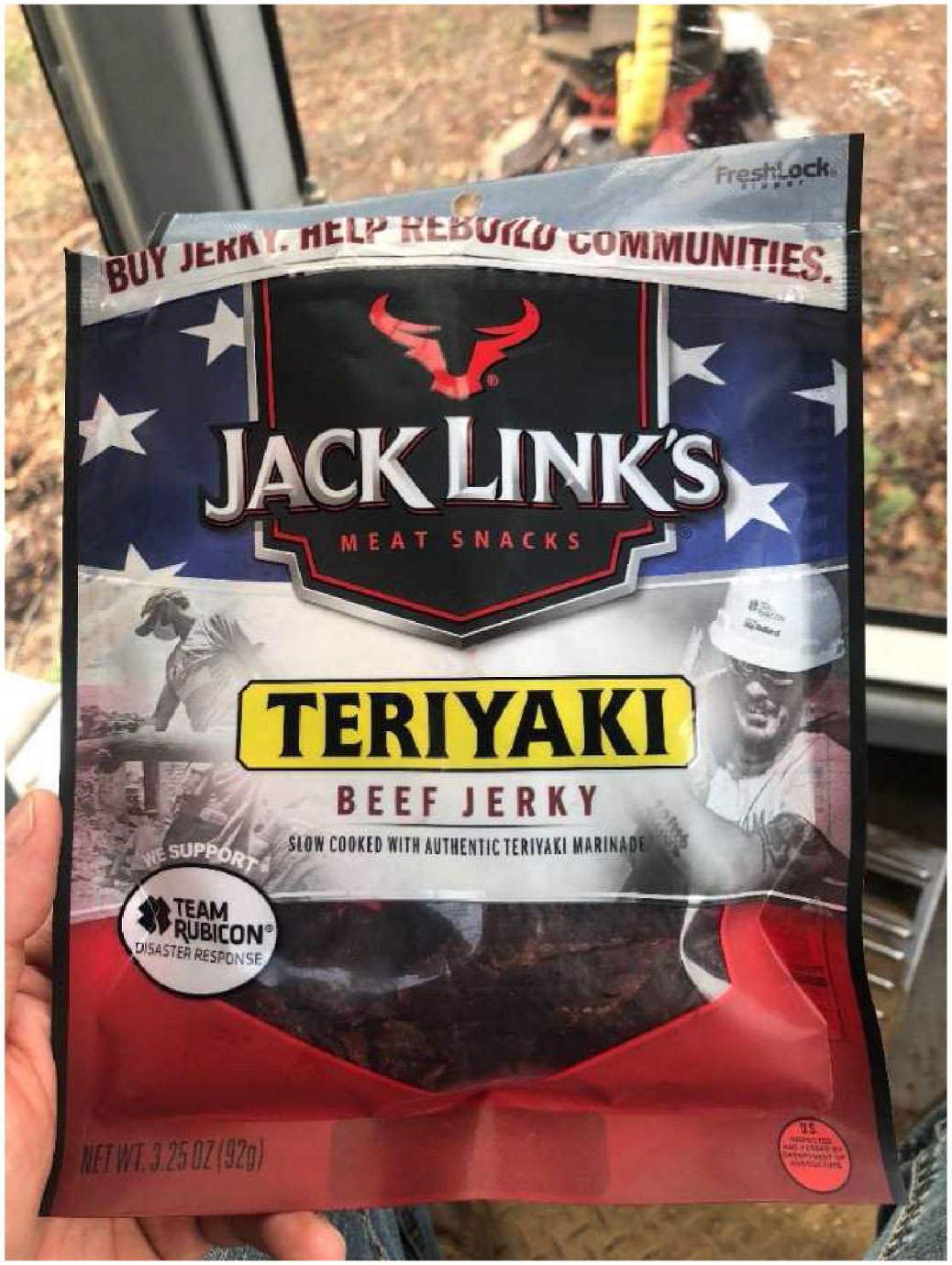
Quote: “When you’re lazy and running late. Plenty of protein and energy. Speaking of energy, I have an energy drink to wash down my jerky. P.S. none of this is good for me or organic” (PID 240).

**Fig. 6.**
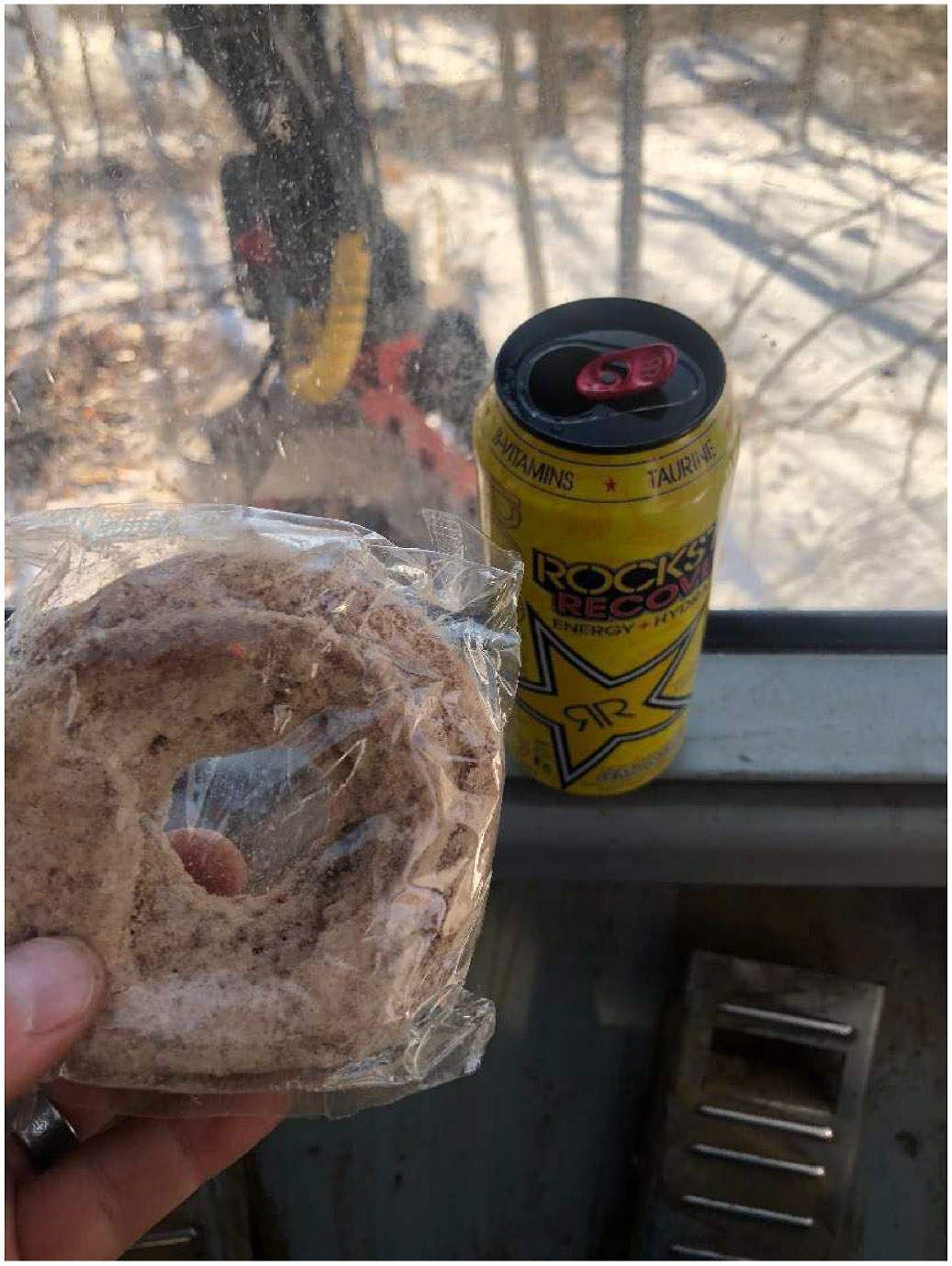
Quote: “Old doughnuts and an energy drink. Definitely not a good combo” (PID 240).

Purchased meals had less variety than homemade, with one participant often consuming energy drinks and pre-packaged muffins or doughnuts for lunch during the workweek (Figs. 5 and 6). Modern processed meat snacks provide protein source but also an abundance of sodium and other chemicals. Energy sources in terms of carbohydrates in the “energy” drink in the photos are extremely limited while caffeine content is very high. The donut provides a limited source of carbohydrates and fat and very little nutrition. Participant that provided the photos in Fig. 6 indicated that he was tired and that eating food makes him tired when he was working. Interestingly, he indicated in his comments that he thought fatigue was one of the most important thing researchers should be looking into for loggers. Based on the photographs he provided, he is most likely not consuming sufficient daily calories for an average man and may be under nourished, at least during the workweek days.

Overall, the homemade meals appeared to have more variety and better quality food over purchased food but still focused heavily on protein and carbohydrates and are lacking in fruit and vegetables, dairy and other fiber and nutrient sources.

### 3.2. Influences in food choice

**Fig. 7.**
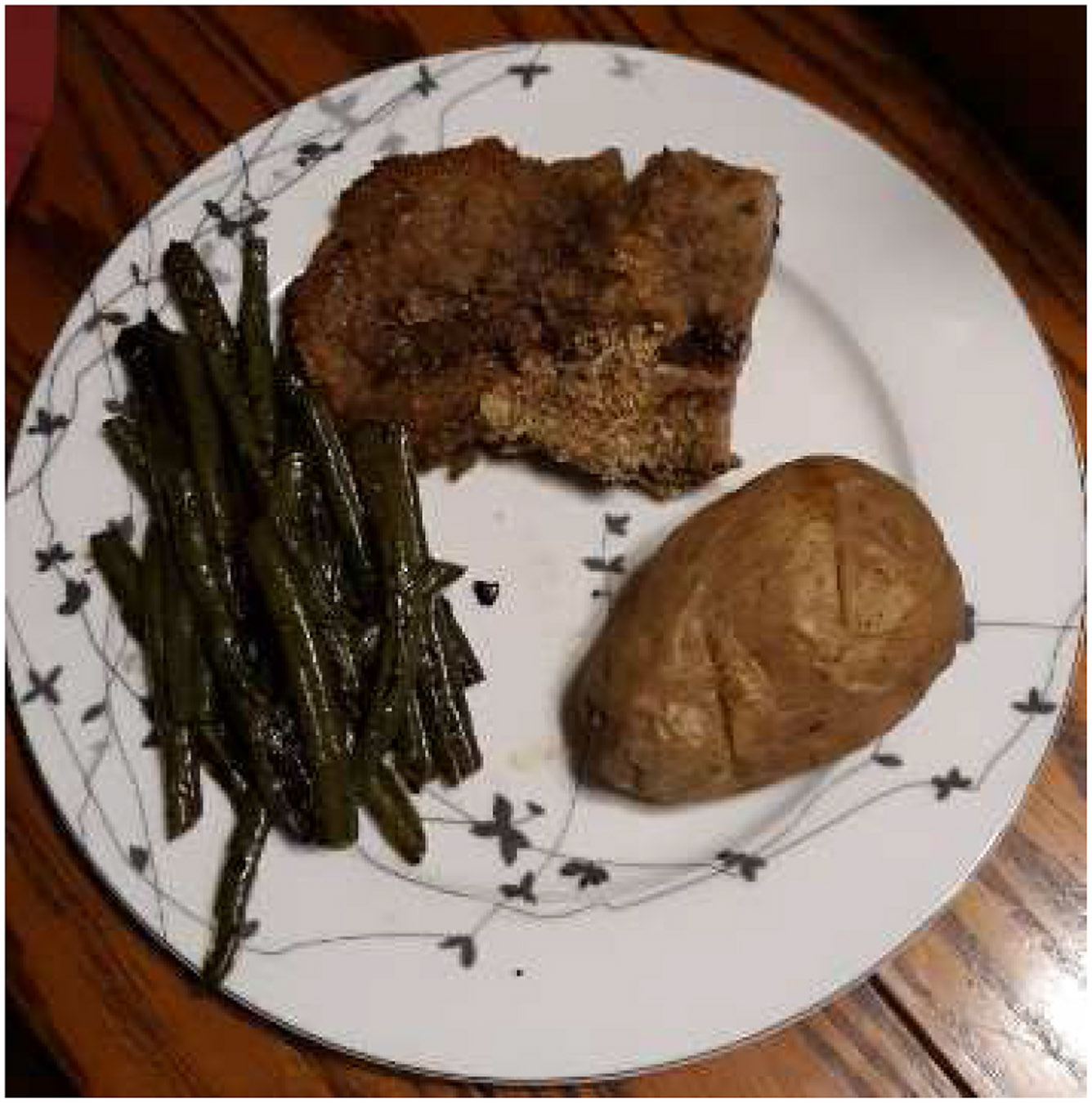
“Pork chops from pigs that I raised. A lot better tasting than store bought and most likely a lot healthier. I fed them a healthy diet and raised them humanely which I think makes the meat a lot healthier than commercially raised pork” (PID 1828.).

In general, there appears to be a distinct difference in attitude towards diet among the six participants. A more positive relationship with food was observed from those that had meals prepared at home. This was especially true for participants who follow a more traditional agrarian style approach to diet, who raised their own meat or vegetables (Fig. 7). This meal consists of three whole foods and includes green beans, baked potato and pork. Although it may be high in fat, nutritional value appears to be higher quality than the pre-made food choices of other participants.

**Fig. 8.**
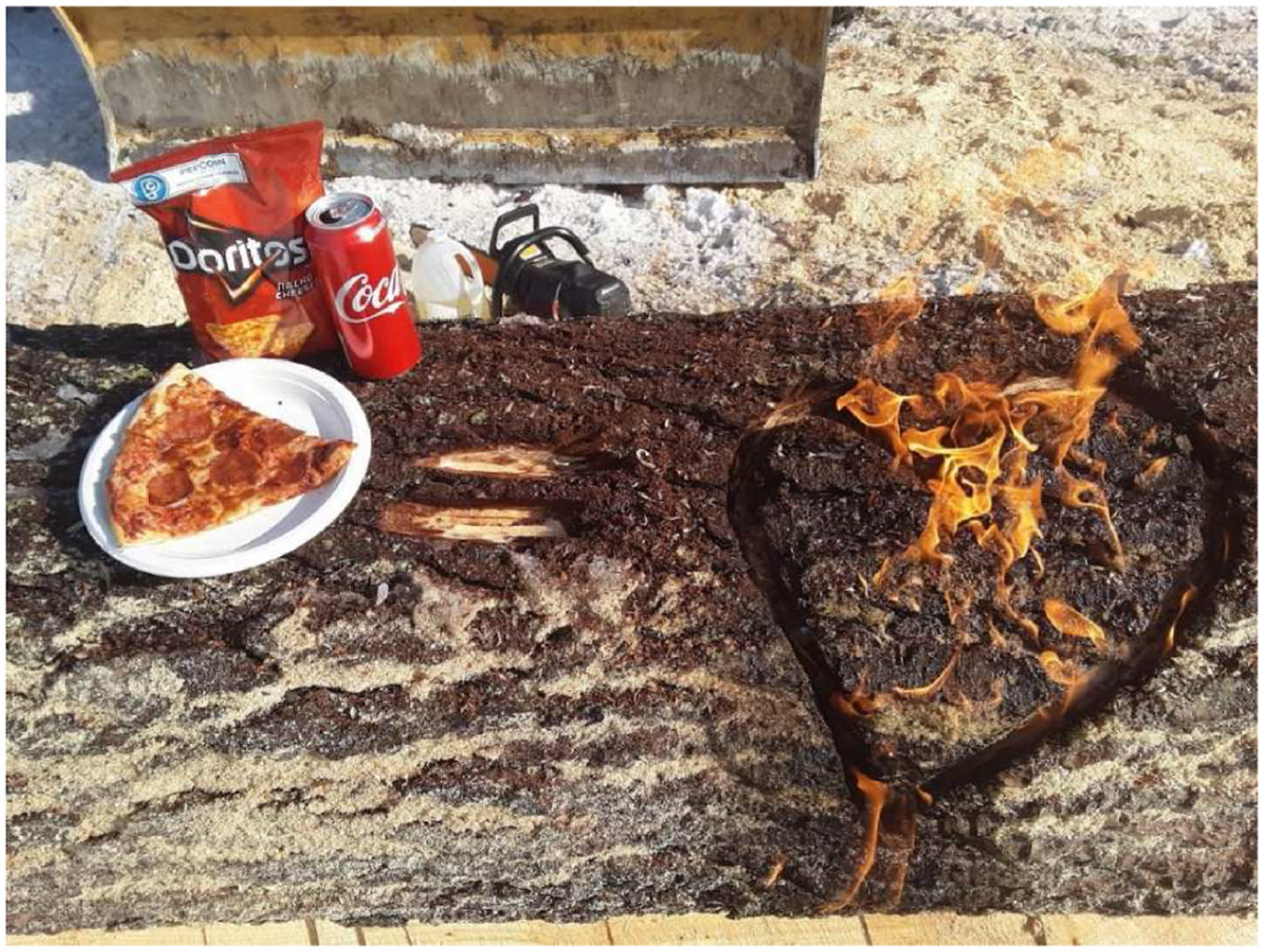
“This is what happens when I go to the local Irving station for diesel at lunch time. Bad Food choices! Again the convenience factor. The heartburn thing is a joke but is also a reality. The foolish thing is, is that I am aware of it. It is just a planning thing” (PID 317).

Negative or apathetic attitudes were expressed towards buying pre-made food at convenience stores linked to convenience and unhealthy diets. One participant said, *“Unfortunately it [bringing food from home] is an inconvenience most of the time. I prioritize by convenience. If there are left overs, that’s convenient, you just pack it up and are good to go. At times, I just go to the store to grab a sandwich which isn’t the most healthy, but again, it’s convenient” (PID 317).* There were multiple comments about loggers not having enough time to make good food choices, e.g. *“… I just don’t have time in the morning to make breakfast. It’s a lot easier to get it at the store” (PID 1828).* Participants acknowledged knowing that store-prepared meals were not healthy, but that expediency was prioritized (Fig. 8). This meal consists of modern American fast food (pepperoni pizza, coke and Doritos) and is highest in fat, salt and carbohydrates, which are contributing not only to indigestion this participant is experiencing but also may contribute to high blood pressure, inflammatory response and cardiovascular disease.

In general, the participants worried about being sedentary and expressed a desire for more free time to enjoy life. When asked what they would do differently or change, loggers expressed a desire to get more exercise, spend more time with family, and cut back on work hours. Some were able to combine quality family time with exercise and food procurement, while hunting with their kids. One participant said, *“Finally a walk in the woods instead of working in the woods! (smiley face emoji). Much needed time with my son!” (PID 1827).*

While many said they desired a healthier diet and lifestyle, they acknowledged barriers to this reality saying, *“I feel like my meals and food priority is very low in comparison to other things. When I plan it, I do eat healthy, but it is not available all the time. The solution is if someone could make an affordable adult size lunchable. Have steak strips and fruit in there, maybe flavored water PID 317.”* Others expressed turning to packaged foods out of laziness or to make up for running late.

Some appeared to use the trip to the local gas station for lunch as a way to take a break from work, saying that *“… I’m sure my employer would rather me bring food from home so I did not have to leave for lunch” (PID 16).* This same participant acknowledged that when he’s working closer to home, he prioritizes working longer hours over grocery shopping and packing his own lunch. Overall, loggers commented about long hours at work, and limited time for recreation, such as hunting, or family time.

### 3.3. Health concerns related to diet

**Fig. 9.**
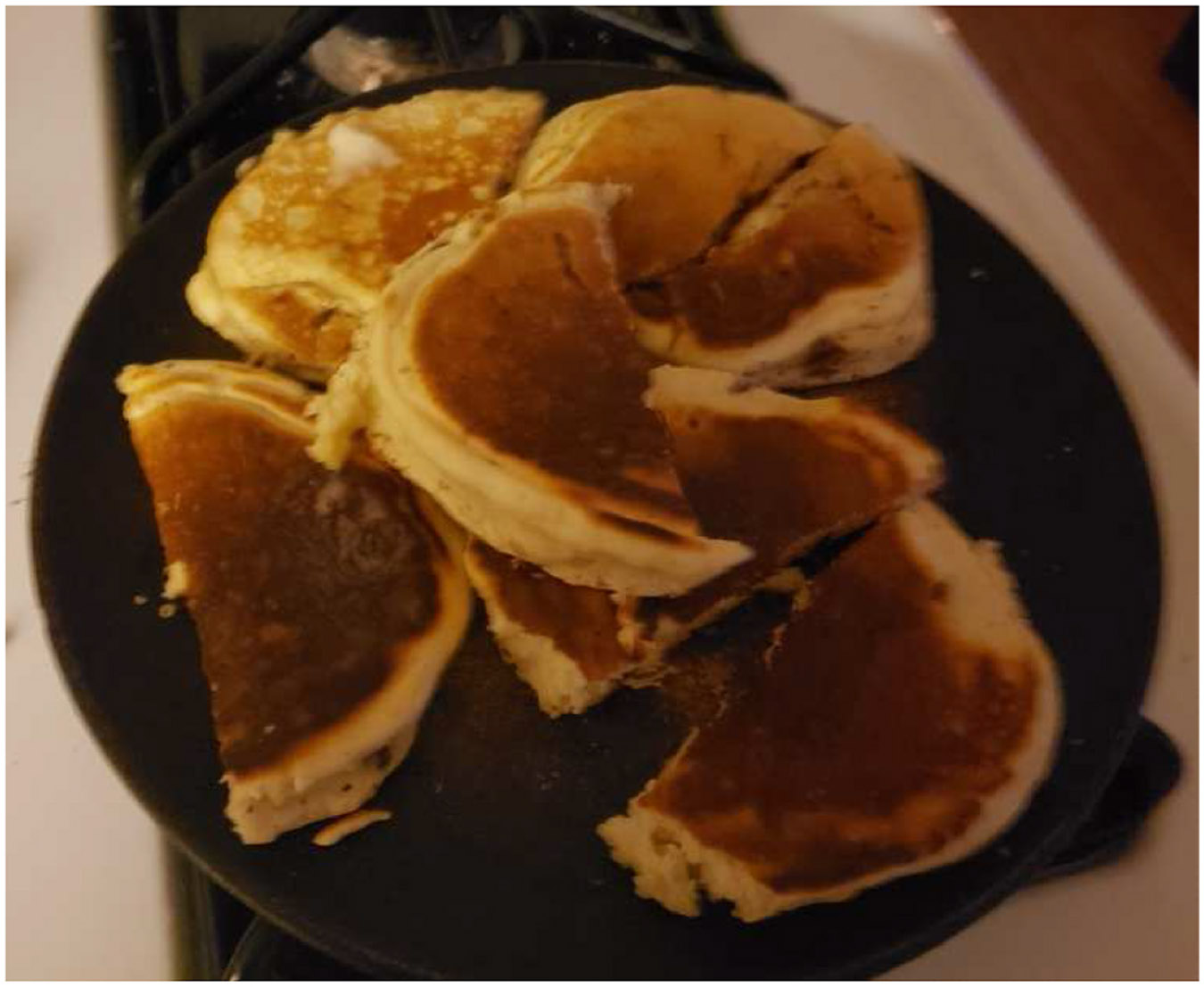
“My wife made pancakes and bacon for the kids! Very tempting but I can’t. Salad and chicken again tonight. (unhappy face emoji). Too many carbs to just sit in a machine.” PID 1827.

Some of the participants talked about health issues, weight, and calorie-restricting diets, with one stating, *“Had my cholesterol checked! Very high no more eggs for breakfast. Two Atkins shakes per day, 1 apple and 1 orange, regular supper” (PID 1827).* It was also difficult to avoid temptations, especially when trying to eat healthfully (Fig. 9). This same participant dealt with feelings of deprivation, saying*, “Oatmeal in the morning does not stick with you for long! Yogurt and rice cake can’t come soon enough!”(PID 1827).*

Another participant complained about fatigue and his diet focused on energy drink consumption. He mentioned that when he was not working he ate breakfast, lunch and dinner at home, but eating at work made him tired. He also expressed concerns that the Photovoice project would just show the *“bad side of people who work in the woods” PID 240.*

In general, the food choices presented in this Photovoice project appeared to be generally very high in portions of protein and processed carbohydrates as well as high in amounts of fat and salt. Basic nutritious food in the form of whole grains, dairy, legumes, plant based proteins and raw or unprocessed vegetables, fish and other seafood do not appear in photographs submitted and most likely missing from diets in general. This lack of variety and the apparent general lack of knowledge or abstention from overall good nutrition is concerning in terms of high blood pressure and cardiovascular health.

## 4. Discussion

This project served to highlight not only the food choices of a small group of loggers, but better understand the barriers and motivators to food choice. Photovoice method was found to be useful in giving a brief but direct voice to loggers on how they feel about their own health and lifestyles. Qualitative analysis also brought out themes between participants and proved as a useful tool in understanding common issues and barriers to healthy eating among this group of people. There were distinct themes of a desire to eat more healthfully but falling short, due to a variety of factors, with time being chief among them. For those who seemed happy with their food choices either 1) they were motivated to plan ahead, or 2) they had a supportive spouse or family who dictated or prepared much of their food. Traditional spousal *roles (“my wife’s good to me” PID 303 or “… you eat what your wife makes you” PID 1828)* and the history of traditional foods of the northeast and Maine and the heritage of logging *(“… I have beans just like the loggers from years ago. You got to [start] the day out right!” PID 303)* seemed to play a role.

Interestingly, the participants who follow a more traditional role in home food preparation and raise animals for meat seemed to demonstrate pride in meal preparation and perceive their food choices as healthy and indeed, the foods prepared did appear to be of higher quality than processed food choices of their counterparts. It may be that traditional hunting and preparing game and raising meat are accepted tasks for the male gender role, whereas more modern grocery shopping, family meal planning and preparation may still generally fall to women. As we saw in one participant’s photos of his children (boy and girl), hunting is taught early and is not exclusively for boys. It is interesting that although many varieties of fruits and vegetables and dairy are available year around in American grocery stores, very few images of fruits and vegetables were seen in the photos in this project, regardless if the men were married or not. Lingering traditions of 19th and 20th century agrarian food production and consumption could have lasting cultural influence for this cohort and their families, especially the practice of preserving foods for winter, where in the past unseasonal fruits and vegetables were not readily available. For those men who are single and do not hunt or raise animals, purchasing a pre-made meal seemed to be an option they exercise more regularly than men with partners and families.

Reviews were mixed on the convenience of store-bought food. It was clear that many priorities came ahead of deciding what to eat, especially earning more income. While some loggers expressed a desire to work fewer hours and spend more time with family, others said that when they get more time in their day, for instance, from a shorter commute to a woodlot closer to home, they work more overtime, enjoying the time saved in commuting. While getting food at the convenience store allowed the worker to take a break from the jobsite, several loggers expressed dismay at how eating “out” all the time was costly. It was clear that some ate their midday meals from the cab of their logging equipment, likely not taking much of a break or moving around. One logger’s comment (PID 1828), *“I just don’t have time in the morning to make breakfast. It’s a lot easier to get it at the store”* highlights the time versus convenience factor. One commented that his employer preferred they did not leave the worksite, presumably because they were more productive. When the loggers’ time is saved, it is a convenience to them, but when the employer’s time is wasted, it is assumed to be seen as an inconvenience from the employers’ perspective. These comments relate to the American lifestyle and issues around productivity, work hours, and work-life balance. Research has shown that workers who take regular breaks, eat well, and get enough rest are ultimately more productive than workers who do not. In the short term, workers may be able to sustain hard work without breaks, but overall, this arrangement results in increased absenteeism and decreased productivity (Grimani et al., 2019). These are concepts that may be difficult for some employers to embrace, especially when, historically, logging embodied the classic American work ethic. It’s possible that the logging industry struggles along with other American industries with the shift from work being focused on material production to identity production (Thompson, 2019). How the individuals view themselves as loggers in context of traditional diets and work habits of the industry may influence their food choices and have an impact on their overall long-term health.

Unlike some literature that discusses food deserts (Ferdinand & Mahata, 2017; Kelli et al., 2017, 2019), this population is very mobile, highlighted by the fact that they all have to commute a far distance for work. It is not a matter of not being able to physically get to a grocery store; rather, it is the time and effort involved to do so. Further, several grow their own food, though the motivation seemed to be the food quality and sense of self-sufficiency rather than the inability to find such items in the grocery store. In addition, while several commented on the cost of convenience store food, there were no comments related to the ability to purchase food, and we attribute this to the fact that they are fully employed workers. In fact, our research has shown that they are relatively financially secure, and we suspect the complaints about cost relate more to the fact that no one wants to spend more money than they have to, over costs being a hardship (Hirabayashi et al., 2021). We suspect that there could be more cultural factors at play, including possible traditional food influences and gendered roles, where it is more common for women to prioritize food shopping, cooking, and packing lunches. We do not know how many of the loggers cook for themselves or feel confident planning and cooking healthy meals, and this is something to explore more fully.

Logging history is rich and varied. Historically, loggers ate the best in the communities in which they worked, which actually became an issue when many Americans were not eating well. Many early cookhouses in Pacific Northwest and eventually in Maine, were revered and claimed to be better than the best restaurants. Meals were varied, plentiful, and made with high-quality foods. Many logging operations developed farms to support feeding loggers, and spared no expense when it came to food. Considered an investment, it was a way to keep men happy and provided an incentive to stay on the job. Fruits and vegetables were always available. While not the Mediterranean diet, it was full of whole foods and fish, fruits and vegetables. Today, loggers are also part of corporate industrial system, but socioeconomics have changed drastically. Loggers tend to be family men and live in towns, and corporate economics, policies, and benefits have changed: loggers have to feed themselves like everyone else.

Once the model of strength and vitality, loggers needed to consume huge numbers of calories (up to 9000 calories a day) to fuel themselves for hard manual labor (Conlin, 1979). Now, with the change to equipment operation, many are concerned about being sedentary, and though they know their work role has changed, some struggle to change their diets to match. One participant indicated his efforts to eat more healthfully when he stated: *“Had my cholesterol checked! Very high no more eggs for breakfast. Two Atkins shakes per day, 1 apple and 1 orange, regular supper” PID 1827.* Although, calorie reduction and weight loss are recommended by medical experts for improving blood pressure and other cardiovascular diseases, meaningful and lasting changes to diet patterns are more important than crash or fad diets. This concern over diet and work balance may be reflective of the particular challenge of how to navigate the cognitive dissonance between the iconic and the modern day logger.

A few of the loggers in the project related to *“the loggers of old”* when referencing a hearty breakfast of beans, sausage and biscuits, also known as the “Great trinity.” Others felt that they could not eat because they have not expended enough energy throughout their workday, even though they report being tired. Moreover, none of them mentioned any physical exercise routines outside of work, an avenue that might be further explored in future work. A few of the participants mentioned families and how that influences diet patterns: 1) want to lose weight, lower cholesterol and be available/around for family and, 2) wives provide support in achieving goals through making meals and shopping. It was evident that many viewed themselves as the image of a family man and provider. The iconic Maine logger and heavy equipment operator of today both need the same quality and variety of food for health and wellbeing. Because of modern cultural norms, and perhaps lingering influences of food traditions that serve as barriers to improved diet patterns, these are struggles that not only loggers deal with but also many Americans. To overcome this involves overcoming an amazing number of barriers: tuning out marketing and advertising, and having know-how to plan, prepare, and deliberate over diet, nutrition and energy needs. While at one time fueling and making these men happy was in the purview of the logging company, today that rests squarely with the loggers themselves, and depending on their circumstances, their families.

### 4.1. Limitations

While the project gives rich insight into a unique group of workers, it was somewhat focused and had relatively few participants. This generated more questions than answers: for example, do individuals really feel guilt or shame about their food choices, or are they proud or indifferent? Does an agrarian state history and traditional food preparation habits influence modern day Maine families? Do Maine logging traditions and folklore still influence how modern day loggers view themselves and have bearing on diets and food choices? In addition, we recognize that participants may have held back on their true feelings, i. e., telling us the socially acceptable response. For example, in the evaluation, one participant acknowledged cropping a candy bar out of the photo - we assume to paint a “healthier” picture for our research team. The themes discussed in this report are generalized to this small group of loggers, though through our own observations and through other quantitative research, we believe they are generalizable to the larger logging community.

## 5. Conclusions

This project served to show that food choice and diet are modulated by complex outside forces, and that improving diet is not a straightforward task. Maine loggers are coping with the same struggles that many workers face, with the added hardship of dealing with extremely long work hours and commutes, leaving little time for anything else. Historic traditions of food preparation and consumption may have a greater influence on modern day diets and diet patterns of this cohort. These factors should be taken into consideration when planning any nutrition-related interventions with logging workers.
